# A Mid-Density Single-Nucleotide Polymorphism Panel for Molecular Applications in Cowpea (*Vigna unguiculata* (L.) Walp)

**DOI:** 10.1155/2024/9912987

**Published:** 2024-01-09

**Authors:** Patrick Obia Ongom, Christian Fatokun, Abou Togola, Ana Luisa Garcia-Oliveira, Eng Hwa Ng, Andrzej Kilian, Stefano Lonardi, Timothy J. Close, Ousmane Boukar

**Affiliations:** ^1^International Institute of Tropical Agriculture (IITA), Kano, Nigeria; ^2^International Institute of Tropical Agriculture (IITA), Ibadan, Nigeria; ^3^International Maize and Wheat Improvement Center (CIMMYT), ICRAF House, UN Avenue, PO Box, Nairobi 1041-00621, Kenya; ^4^Department of Molecular Biology, College of Biotechnology, CCS Haryana Agricultural University, Hisar, India; ^5^Excellence in Breeding Platform, International Maize and Wheat Improvement Center (CIMMYT), Los Baños, Laguna 4031, Philippines; ^6^Diversity Arrays Technology Pty Ltd., University of Canberra, Montana St., Bruce, ACT 2617, Australia; ^7^Department of Computer Science and Engineering, University of California, 900 University Avenue, Riverside, CA 92521, USA; ^8^Department of Botany and Plant Sciences, University of California, 900 University Avenue, Riverside, CA 92521, USA

## Abstract

Molecular markers are increasingly being deployed to accelerate genetic gain in crop plants. The objective of this study was to assess the potential of a mid-density genotyping panel for molecular applications in cowpea breeding. A core set of 2,602 targeted diversity array technology (DArTag) single-nucleotide polymorphisms (SNPs) was designed from an existing 51,128 Cowpea iSelect Consortium Array. The panel's usefulness was assessed using 376 genotypes from different populations of known genetic backgrounds. The panel was informative, with over 78% of SNPs exceeding a minor allele frequency of 0.20. The panel decoded three stratifications in the constituted population, as was expected. Linkage disequilibrium (LD) decay was correctly depicted as slower in a biparental subset than in other populations. A known flower and seed coat color gene region was located on chromosome Vu07, suggesting that the mid-density panel may be used to hypothesize genomic regions underlying target traits in cowpea. Unexpected heterozygosity was detected in some lines and highly among F_1_ progenies, divulging the panel's potential application in germplasm purity and hybridity verification. The study unveils the potential of an excellent genomic resource that can be tapped to enhance the development of improved cowpea cultivars.

## 1. Introduction

Cowpea (*Vigna unguiculata* (L.) Walp) is a diploid species (2*n* = 2 × = 22) with a genome size estimated at 640.6 Mbp based on cytometry [1]. Cowpea is globally recognized as a key food and nutritional security legume in sub-Saharan Africa (SSA). It is grown in areas regarded as marginal for many other crops owing to its relative inherent drought and heat tolerance and ability to fix soil nitrogen [2]. Cowpea feeds more than 200 million people in SSA, where the major producers are smallholder farmers, and the crop is often grown as an intercrop with cereals [2, 3]. Farmers grow this protein-rich crop for its grains, tender leaves, and pods, consumed as food, while the crop residues are used for fodder or added back to the soil to improve fertility [4]. Despite its significance, cowpea suffers yield penalties from several biotic and abiotic stresses, including diseases (bacterial, fungal, and viral), insect pests, parasitic weeds, and severe drought and heat [5–7]. These challenges can be mitigated through the development and deployment of improved varieties that are stress resilient.

Given the strategic placement as a food security crop, cowpea is gaining more research attention globally. Such efforts have, in the past few years, led to the development of excellent genetic resources that are being tapped to improve the crop's productivity. For instance, the International Institute of Tropical Agriculture (IITA) maintains over 15,000 accessions of cowpea from which the core and minicore subsets representing global diversity have been constituted [8]. In addition, different genetic resources have been developed at IITA and collaborative institutions including Multiparent Advanced Generation Intercross (MAGIC) population developed by the University of California Riverside (UCR) [9], biparental recombinant inbred lines (RILs) [10], and many elite breeding lines developed by IITA [2, 11]. Another key genetic resource for cowpea is the UCR minicore, consisting of 368 worldwide accessions of cultivated cowpea [12]. Further, the United States Department of Agriculture (USDA) holds over 7,525 cowpea accessions, from which a subset of 700 accessions were constituted for genetic exploitations [13, 14]. These resources have been tapped for trait improvement by several cowpea breeding programs across the world.

Efforts to accelerate genetic gain in cowpea have also led to the development of genomic resources. The genome of cowpea has been dissected beginning with a single reference genome based on IITA line 1T97K-499-35 [1], currently expanded to seven reference genomes termed as the pangenome of domesticated cowpea (https://phytozome-next.jgi.doe.gov/cowpeapan). Genotyping platforms for cowpea germplasm have also been developed to exploit best these genomes and broad genetic diversity within cowpea germplasm. The first was the 1536-SNP GoldenGate assay [10], which has been used for linkage mapping and QTL analyses [15–17] and assessment of genetic diversity [18]. The IITA minicore has also been genotyped based on genotyping-by-sequencing (GBS) using 2,276 SNP markers to allow practical utilization of the germplasm [8]. Another platform with high-density markers is the Illumina Cowpea iSelect Consortium Array, which represents a publicly accessible resource for screening 51,128 single-nucleotide polymorphisms (SNPs) [19]. Due to cost limitations associated with these platforms, the focus has recently shifted to using reduced-cost genotyping methods. For instance, Wu et al. [20] developed a Kompetitive Allele-Specific PCR (KASP) assay for cowpea; however, because of the relatively high cost of the KASP assay, only 50 informative SNPs were recommended with limited usage. Compared to the genotyping platforms cited above, diversity array technology (DArT) has been described as a low-cost, high-throughput, robust system with minimal DNA sample requirements capable of providing comprehensive genome coverage even in organisms without any prior DNA sequence information [21]. Since its invention, the DArT platform has been extensively utilized in various crops, including cowpea, for different purposes: QTL mapping for grain yield traits using the DArTseq platform [22], genetic diversity, and population structure analysis using DArTseq SNPs [23, 24].

DArT has evolved, leading to multiple options tailored to specific breeding needs. Among the suites of DArT options that have recently been developed is the targeted genotyping (DArTag) method, which allows genotyping using selected marker sets (https://www.diversityarrays.com/technology-and-resources/targeted-genotyping/). DArTag is a variant of many of the targeted genotyping suites developed by the DArT company. With DArTag, any SNP (or a small indel) can be targeted if some genomic sequence is available around the variant base/indel. DArTag offers cost efficiency and reduced bioinformatics load, well suited for high-throughput scenarios.

In the present study, we validated the usefulness of a medium-density DArTag marker panel for cowpea and demonstrated its potential application for genetic studies and utilization in molecular breeding. This genotyping panel has a core set of 2,602 SNPs, custom-designed from a publicly available 51,128-SNP Cowpea iSelect Consortium Array obtained from Muñoz-Amatriaín et al. [19]. The specific objective was to assess the performance of this custom-made SNP panel in diversity studies, population structure characterization, trait mapping, and potential applications in quality control (QC).

## 2. Materials and Methods

### 2.1. Plant Materials

The genetic materials used in this study were constituted from groups of cowpea genotypes having different genetic backgrounds. The genetic groups included elite breeding lines, germplasm accessions from the IITA Genetic Resources Center, and multi- and biparental recombinant inbreds, making 376 genotypes (Table 1). The first group consisted of 123 elite breeding lines from IITA that are generally used as parents in several cowpea breeding programs. These lines are high yielding, drought tolerant, heat tolerant, and striga resistant and have several seed quality traits demanded by farmers in SSA. The second category included 22 accessions selected from the IITA cowpea minicore population. The cowpea minicore is a subset of a world cowpea germplasm collection maintained at the IITA crop genetic resource gene bank, and they are good sources for traits of economic importance in cowpea [8, 25]. The third group consisted of 100 MAGIC inbred lines previously described by Huynh et al. [9], here on referred to as multiparental RILs. These RILs combine many abiotic and biotic stress resistances, seed quality, and agronomic traits relevant to cowpea in SSA. A fourth group was a random sample of 101 biparental RILs derived from a cross between aphid-resistant wild relative TVNu1158 and elite IITA line IT99K-573-1-1. The fifth category included 30 F_1_ progenies derived from different crosses in the breeding program, mainly to help verify the marker panel's sensitivity in differentiating between heterozygous and homozygous genotypes.

### 2.2. Sample Preparation

The 376 cowpea genotypes were planted in the screenhouse (latitude 11°58′51.5^″^N, longitude 8°33′28.3^″^E) in pots of size 24 cm (height) × 25.4 cm (diameter), three-quarters filled with sterilized topsoil, placed on the crossing benches. Three seeds were sown per pot and thinned to one seedling a week after emergence. Two weeks after, a young trifoliate leaf from each plant was sampled for DNA analysis. The sampling was done according to the procedure described by Intertek-Agritech laboratory [26]. A more detailed sampling procedure has been described by Ongom et al. [27].

### 2.3. DNA Isolation and Genotyping

Total genomic DNA was isolated at the Intertek Laboratory, Australia, and the samples were forwarded to Diversity Arrays Technology (DArT) facility for genotyping. Genotyping was done by employing DArTag technology, one of the targeted genotyping approaches which offers the capacity to genotype materials using specific or selected sets of SNP markers (https://www.diversityarrays.com/technology-and-resources/targeted-genotyping/). For the 376 leaf samples, a panel of 2,602 SNP markers regarded as the *Cowpea mid-density genotyping panel V1.0*. was used. These markers are a subset from the 51,128-SNP Cowpea iSelect Consortium Array [19] and were selected based on iSelect data from 2,714 diverse cultivated cowpea accessions, with extra weight given to 184 accessions most used in African breeding programs. The criteria used for marker selection were (1) iSelect missing data rate less than 5%, (2) iSelect data MnAF > 0.2, and (3) even spacing along the genetic linkage. The SNPs matching these criteria were included in a DArTag test set against 376 cowpea DNA samples (Supplementary Table 1). DArTag genotyping was accomplished using special molecular probes that select the small target regions containing sequence variants. The targeted regions were then amplified and, in parallel, the sample-specific barcode was attached. The libraries generated were sequenced on the next generation sequencing (NGS) equipment, Illumina Hiseq2500/Novaseq with 1,200,000 reads per sample. The resulting sequences were processed using DArT PL's proprietary pipeline that includes sequence alignment to sequences matching fragments of the IITA cowpea IT97K-499-35 reference genome [1] *Vigna unguiculata* v1.1, publicly accessible on Phytozome (https://phytozome-next.jgi.doe.gov/info/Vunguiculata_v1_1) delineated by the DArTag oligos from the panel and allele calling based on counts of alternative alleles for each sample and marker.

### 2.4. Data Filtering

The data received from the DArT facility contained 362 out of 376 cowpea genotypes which included both the F_1_ progenies and the lines. DArT report was not generated for 14 genotypes due to extreme missing data. Upon receipt, data were filtered using TASSEL v.5.2.79 [28] for missingness and low minor allele frequency (MnAF) with the following criteria: SNPs with >20% missing data and MnAF < 0.05 were removed, leaving 2,435 SNPs. This data set was then used to test the marker panel for application in breeding as quality control (QC) markers. In the second filtering step, the 30 F_1_s were excluded from the data set and the remaining data were filtered against high heterozygosity, where genotypes with >0.3 heterozygosity were removed. The resulting data, consisting of 2,230 SNPs and 330 cowpea genotypes, underwent LD pruning using the function *snpgdsLDpruning*() in SNPRelate package [29], and the LD threshold was set at 0.2. This generated 871 pruned SNPs that were in LD equilibrium. The raw SNP data set has been deposited in the European Nucleotide Archive (ENA) with reference number PRJEB56743 (ERP141707).

### 2.5. Statistical Analysis

#### 2.5.1. SNP Polymorphism and Distribution

Frequencies of minor alleles, major alleles, heterozygosity, missing data, and SNP summary information were generated in TASEL v.5.2.79. These records were used to generate the distributions of allele frequencies and SNP density plots in R using *ggplot2* package.

#### 2.5.2. Population Structure Analysis

Pruned SNP data were formatted for structure analysis utilizing TASSEL v.5.2.79 and PGDSpider v.2.1.1.5 [30]. Structure analysis was performed using STRUCTURE 2.3.4 [31]. Parameters were configured and set to 5,000 Burnin period, while the number of Markov chain Monte Carlo (MCMC) repetitions was 50,000, and the admixture model was chosen. A simulation was then implemented, setting the number of assumed populations (*K*) from 1 to 10 and with 20 iterations for each *K*. Results were summarized using STRUCTURE Harvester, Web v0.6.94 [32] following the method described by Evanno et al. [33]. In addition, replicated results from structure program were summarized using CLUMPP (Cluster Matching and Permutation Program) version 1.1.2 [34]. The resulting output from CLUMPP was then used in software DISTRUCT version 1.1 [35] to generate a graphical visualization of the population structure. Further, PCA was conducted in R using the filtered and imputed SNPs. Missing data was imputed using *missMDA R package*, based on the *K*-fold method [36] and visualized using factoextra package [37]. In addition, hierarchical cluster analysis of the population was conducted using the *pheatmap* package.

#### 2.5.3. Population Differentiation Analysis

Differentiation statistics and analysis of molecular variance (AMOVA) were used to compare diversity within and between four genetic populations, after excluding the F_1_ progenies. Analysis of molecular variance was conducted using *poppr* package. Measures of population differentiation (*F*_ST_ and *G*_ST_) [38] were generated using *mmod* R package. Gene flow (*N*_*m*_) was estimated from *F*_ST_ according to the island model [39, 40] as follows:  *N*_*m*_ ≈ 0.25(1 − *F*_ST_)/*F*_ST_.

Discriminant analysis of principal components (DAPC) was conducted using *adegenet* package to check the structure within the populations [41]. The number of PCs retained in DAPC was set to 100 after inspecting the curve of variance explained by PCA while the number of discriminant clusters was determined using the Bayesian information criterion (BIC) method [41].

#### 2.5.4. Linkage Disequilibrium Analysis

We estimated the rate of LD decay in the four cowpea genetic populations. A measure of LD (*r*^2^) and pairwise distance between SNPs were generated in TASSEL v.5.2.79 and the rate of decay on each of the 11 cowpea chromosomes was visualized with graphics generated with *ggplot2* package in R. Mean LD per chromosome was calculated after every 0.5 Mb interval, and the average genome-wide decay rate estimated by averaging LD in each interval across all chromosomes. LD was also computed for each of the four genetic populations separately to decode the difference in the decay rate within each population and the entire population. A line graph was used to display the overlay of chromosome and population-specific LD and the mean genome-wide LD decay rates.

#### 2.5.5. Trait Mapping Potential

Despite the medium size of the SNP panel, we performed a combined genome-wide scan and linkage mapping using existing flower and seed color phenotypes to test the possibility of its deployment in generating clues regarding genomic regions that might be associated with traits of interest. The two traits also contrasted the parents of the sampled BPRs, IT99K-573-1-1 (white flower, white seed coat) × TVNu1158 (purple flower, speckled seed coat). A genome-wide scan was performed using all 330 lines, followed by linkage mapping using the biparental RILs alone. The populations were planted at the IITA Minjibir research farm in Kano State, Nigeria (12.1924°N, 8.6284°E). The nursery was established with 1-meter, single-row plots arranged in an augmented design. At the reproductive stage, flower colors were scored visually and later encoded into numeric values. The two major flower colors exhibited in the population were white and purple, scored 1 and 0, respectively. Similarly, after harvest, major seed colors were identified, recorded visually, and encoded into numeric values: white = 1, brown = 2, black = 3, purple = 4, speckled = 5, and mosaic colors = 6. Linkage analysis was performed using QTL IciMapping V4.2 software using the MAP function [42]. Before linkage map construction, markers with significant segregation distortion (*P* ≤ 0.05) and redundant markers were excluded. Marker distances and orders were based on the Kosambi map function, and the mapping of the quantitative trait loci (QTLs) was performed using the inclusive composite interval mapping (ICIM-ADD) function for the two traits investigated in the present study. To declare a significant main effect QTL, a LOD threshold was set at 3.0. QTLs explaining phenotypic variation (PVE) ≥ 10% were considered major QTLs, while below this value were minor QTLs.

#### 2.5.6. Quality Control

To evaluate the application of the panel for quality control/quality assurance (QC/QA) in breeding programs, we compared heterozygosity level of individual genotypes from the different germplasm categories included in the study: breeding lines, F_1_ progeny, accessions, multi- and biparental RILs. TASSEL v.5.2.79 was used to compute proportion of heterozygous loci for each cowpea genotype. Box plots and a faceted dot graph were generated in R to depict the distribution of heterozygosity for the different categories of cowpea genotypes included in the sample. In addition, the 30 F_1_s and their parents were considered separately for hybridity analysis. First, cluster analysis was conducted to determine diversity among the parents of F1s. For this analysis, a neighbor-joining method [43] was used to generate the genetic distances, and a cladogram using archaeopteryx in TASSEL v.5.2.79 was used to visualize the clustering among parents. This was followed by an analysis of marker polymorphism between every pair of parents used in making the 30 F_1_s as previously described [27]. Markers found to be polymorphic between the parental pairs were then used to assess the level of hybridity among the F_1_s. Hybridity was expressed as a ratio of the number of polymorphic markers that detected a particular F_1_ as being heterozygous to the total number of polymorphic markers between the parents of that cross [27]. Further, SNP marker efficiency was assessed by determining how frequent a marker was polymorphic across the 30 pairs of parents [27].

## 3. Results

### 3.1. Polymorphisms

**Figure 1:**
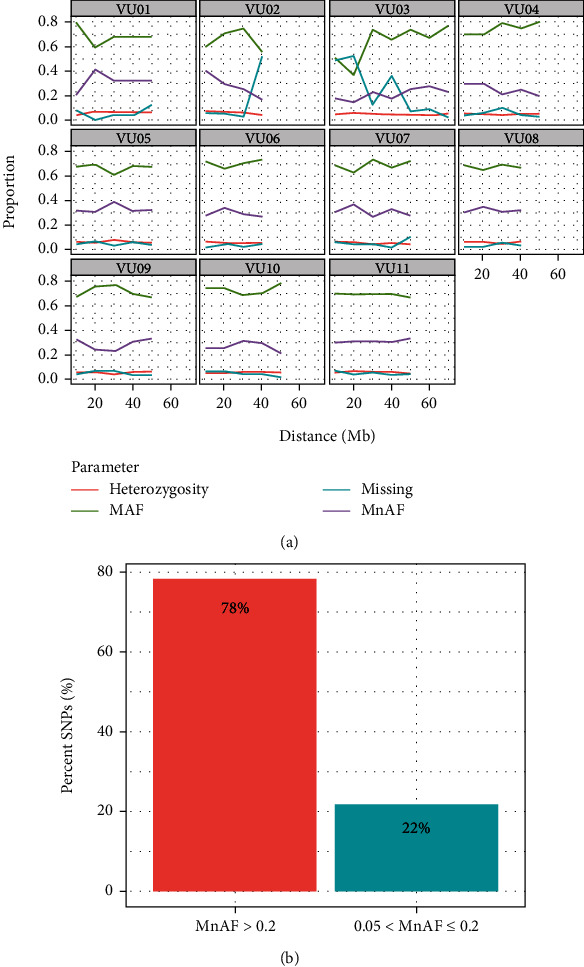
Distribution of allele frequency. (a) Chromosome-wide distribution of major and minor allele frequency proportions, including proportion of heterozygous loci and missing data. (b) Percentage distribution of informative SNP markers as defined by proportion of minor allele frequency (MnAF) above and below 0.2.

We examined the informativeness of the marker panel based on the heterozygosity of loci, allele frequencies, and nucleotide density. Chromosome-wide distribution of allele frequencies and heterozygosity are presented in Figure 1(a). These genetic parameters varied along chromosomes but generally exhibited high major allele frequency followed by minor allele frequency, while heterozygosity proportions remained low across all chromosomes. The proportion of missing marker data was also generally low, except on chromosomes Vu02 and Vu03, where the regions at 40 Mb and 20 Mb, respectively, had high missing data. The mean proportion of heterozygous loci ranged from 0.047 on chromosome Vu03 to 0.065 on chromosome Vu05, with a chromosome-wide average of 0.056 (Supplementary Table 2). The mean major allele frequency ranged from 0.64 on chromosome Vu03 to 0.75 on chromosome VU04, with a genome-wide average of 0.69. Meanwhile, minor allele frequency ranged from 0.21 on chromosome Vu03 to 0.33 on chromosome Vu05 and a chromosome-wide average of 0.29 (Supplementary Table 2). Overall, 78% of SNPs had minor allele frequency above 0.2, while the remaining 22% had allele frequencies that were ≤0.2 but still above 0.05 (Figure 1(b)).

**Figure 2:**
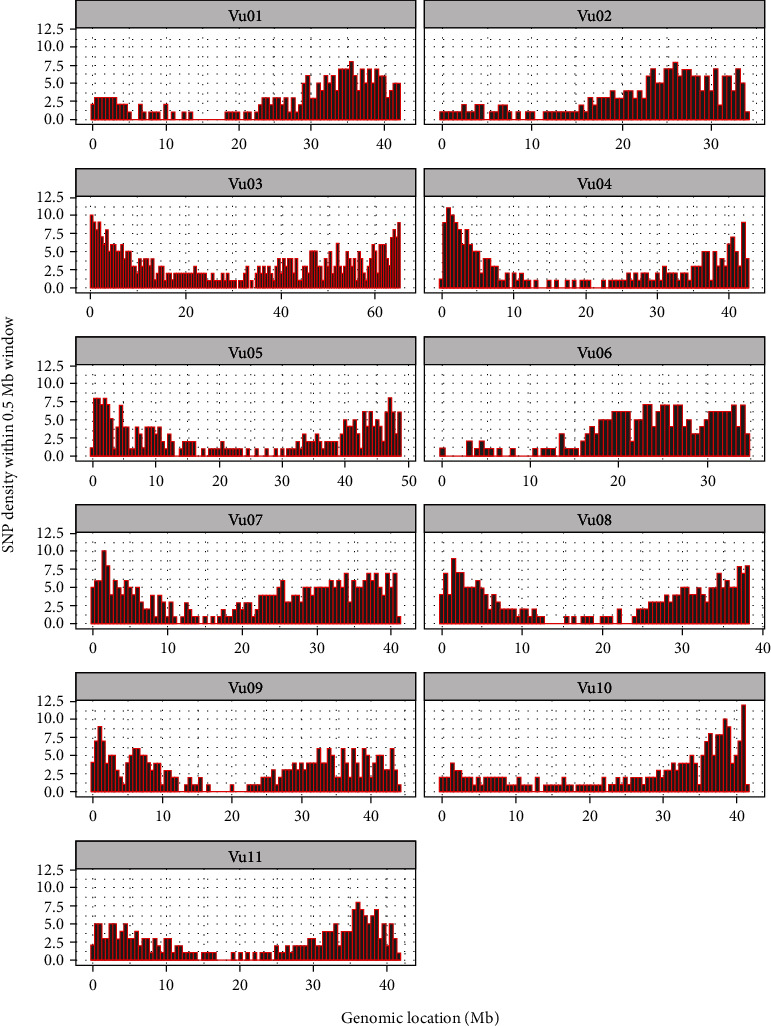
Chromosome-wide distribution of SNPs along the 11 chromosomes of cowpea (*Vigna unguiculata*). Data on the number of SNPs per chromosome is provided in supplementary Table 2.

The distribution of SNPs per chromosome based on the number of SNPs within 0.5 Mb window size is presented in Figure 2. Chromosome lengths varied with the shortest and longest chromosomes being Vu02 (33.75 Mb long) and Vu03 (64.99 Mb long), respectively (Figure 2 and Supplementary Table 3). Chromosome Vu03 had the highest number of SNPs (295), while chromosome Vu10 had the lowest number of SNPs (165), with a genome-wide average number of SNPs per chromosome being 202.73 (Supplementary Table 3). Considering the varying chromosome lengths, chromosome Vu07, which harbors 256 SNPs, registered the highest SNP density of 6 SNPs per Mb. In comparison, Vu05 had the lowest density of approximately 4 SNPs per Mb and a chromosome-wide average SNP density of 4.79 SNPs per Mb. Consequently, the chromosome-wide average distance between SNPs was estimated at 0.2 Mb (i.e., one SNP every 200Kb), with a range of 0.16 Mb on chromosome Vu07 to 0.26 Mb on chromosome Vu05 (Supplementary Table 3).

### 3.2. Population Structure

**Figure 3:**
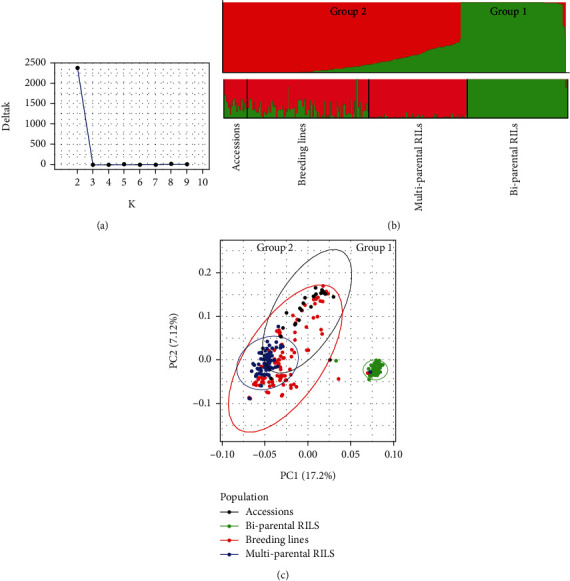
Population structure of the 330 cowpea genotypes constituted from a sample of lines coming from four different genetic backgrounds. (a) Plot of *K* versus *DeltaK* showing the most probable number of subgroupings (*K* = 2). (b) STRUCTURE bar plots depicting two groups before excluding bparental recombinant inbred lines (RILs); Supplementary Figure 2 shows the exposition of additional subgrouping in the remaining data set after dropping biparental RILs. (c) Principal component analysis (PCA) displaying the scattering of genotypes along X (PC1) and Y(PC2) axes. PCA revealed a close scattering of biparental RILs, with the accessions and breeding lines together being the most scattered, while multiparental RILs are clearly visible as a moderately clustered subset within the bigger group.

Structure analysis revealed that the most probable number of subgroupings when all the 330 genotypes were considered together was *K* = 2 as depicted by the *DeltaK* vs. *K* plot (Figure 3(a)), complemented by group assignment depicted by the STRUCTURE bar plots in Figure 3(b). It was evident from these bar plots that the biparental RILs were assigned to one group while the rest of the genotypes formed a second large and diverse group. Further investigation based on the probability of group assignment revealed that subgroup one was made up of 30% of the total population, and out of this, 97% were purely the biparental RILs; the remaining 3% of group one consisted of 2 breeding lines and 1 multiparental line (Supplementary Table 4). The second subgroup constituted 59% of the population, and it contained about 90 breeding lines, 94 multiparental RILs, and 11 accessions. The remaining 11% were those that were categorized as admixed and were made up of 11 accessions and 26 breeding lines (Supplementary Table 4). A heatmap showing the relationship among the four genetic populations and how they fit in the STRUCTURE inferred groups is presented in Supplementary Figure 1A. The heatmap overlaid the four genetic populations on the group assignments inferred by STRUCTURE software, revealing that all biparental RILs belong to group 1 of the STRUCTURE inference while group 2 harbors the remaining three populations. Similar results were depicted by the dendrogram presented in Supplementary Figure 1B.

However, the fact that biparental RILs were completely dissociated from the rest of the population, it was suspected that this could introduce confounding effects in the STRUCTURE results. Indeed, excluding biparental RILs from the analysis revealed additional stratification in the remaining data set (Supplementary Figure 2A and B). That is, two groups were detectable after excluding the bi-parental RILs, with group 1 having a total of 40 lines, 58% of which were breeding lines, 43% were the accessions, yet the multiparental RILs had zero membership in this group (Supplementary Figure 2C). Group 2 was the largest with 158 lines, 52% being multiparental RILs, and 47% were the breeding lines, while only one accession was a member of this group. A total of 36 lines were categorized as admixed since they had an almost equal probability of belonging to both groups. Similar obscurity in population structure was depicted by PCA when the entire data set was considered, portraying biparental RILs as forming a single group while the other three genetic populations, together, formed a second group (Figure 3(c)). The PCA further revealed that within group 2, the breeding lines and accessions were the most scattered, while multiparental lines were closer together (Figure 3(c)). These observations suggested three subgroups in this population, but a clear separation was confounded by the presence of the bi-parental RILs in the data set.

**Figure 4:**
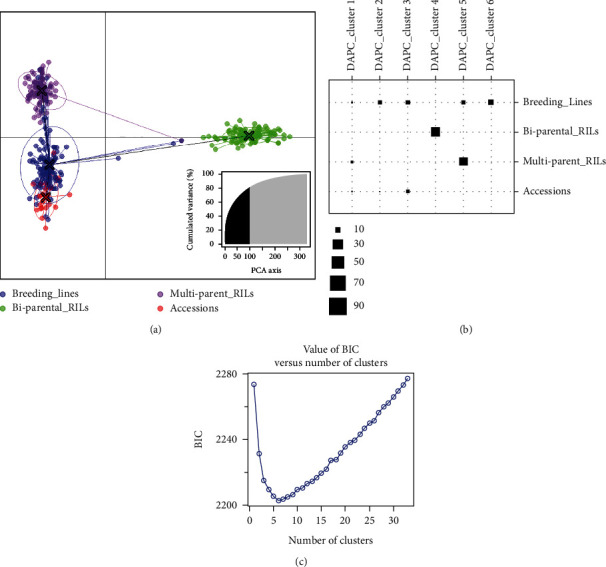
Discriminant analysis of principal components (DAPC). (a) DAPC depicting a clear differentiation between biparental RILs and the other three populations but precisely separated multiparental RILs from breeding lines while accessions remained together with the breeding lines. A clear separation of the population into three gene pools is depicted by DAPC. (b) DAPC clustering showing the extent of structure within each genetic population: breeding lines being the most structured, having multiple black blocks, and biparental RILs being the least structured, having just one black block; legend at the bottom of (b) indicates the relative sizes of the blocks. (c) Bayesian information criteria (BIC) versus the number of clusters, depicting appropriate number of clusters used in determining extent of structure within each genetic population. The optimal number of clusters corresponds to the lowest point of the curve where BIC is at its lowest value.

Interestingly, this confounding structure was powerfully revealed by discriminant analysis of principal components (DAPC) (Figure 4(a)). In this analysis, biparental RILs were distant from the rest of the populations, yet there was a clear separation between the multiparental RILs and breeding lines, while the accessions remained closer to the breeding lines. Clearly, there were three groups in the population (Figure 4(a)). A further investigation of discriminant clusters revealed that breeding lines were the most stratified and diverse, followed by the accessions, the multiparental RILs, and the biparental RILs were the least structured (Figure 4(b)). Bayesian information criteria (BIC) plot from DAPC analysis detected six clusters in the population which were used in determining the extent of the structure or diversity within each genetic population (Figure 4(c)). The BIC plot supported the extent of scattering observed in Figure 4(b).

### 3.3. Population Differentiation

To gauge how well the SNP panel can discern the differentiation between and within populations, we computed the pairwise genetic distances between populations, followed by an analysis of molecular variance (AMOVA). There was clear differentiation among the genetic populations. Genetic distance and differentiation measures ranged from Dist. = 8.38, *F*_ST_ = 0.06, and *G*_ST_ = 0.04 to Dist. = 22.26, *F*_ST_ = 0.41, and *G*_ST_ = 0.27, with the low values recorded among breeding lines, multiparental RILs, and accessions while high values were registered when biparental RILs were compared with the rest of the genetic populations (Table 2). Pairwise gene flow (*N*_*m*_) among the four genetic populations ranged from *N*_*m*_ = 0.36 (biparental RILs vs. accessions) to *N*_*m*_ = 3.92 (breeding lines vs. multiparental RILs) and mean of *N*_*m*_ = 1.3 (Table 2). Overall, low gene flow estimates were registered between biparental RILs and all other genetic populations, a pattern that interestingly corresponded with high differentiation measures (Table 2).

AMOVA (Table 3) revealed significant genetic variations among genetic populations (*P* = 0.01) while variation between the populations was not significant (*P* = 0.18). The overall variation among genotypes across all four populations was highly significant (*P* = 0.01). Variation among populations accounted for 16.64% of total variation, while that between populations accounted for only 8.38%, and variability among all genotypes across populations accounted for 74.98%. Population differentiation statistic (phi) was similarly higher among genetic populations (phi = 0.18) and genotypes across all populations (phi = 0.25) compared to between population variations (phi = 0.08).

### 3.4. Linkage Disequilibrium Decay and Trait Mapping

**Figure 5:**
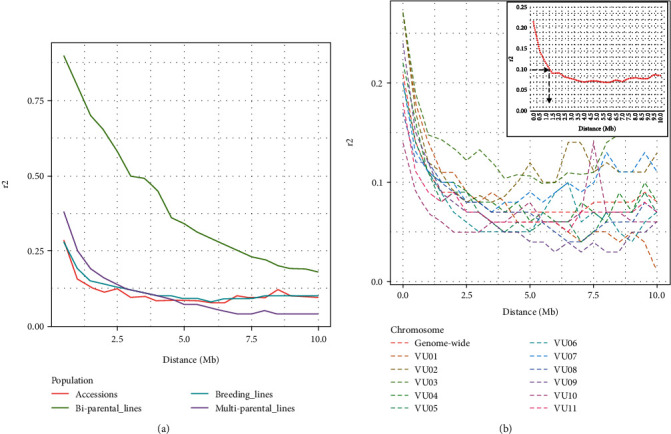
Linkage disequilibrium decay (LD) in a constituted population of cowpea. (a) LD decay within four genetic populations of cowpea: biparental RILs (slowest decay rate), multiparental RILs, breeding lines and accessions (fastest decay rate). (b) Chromosome-wide LD decay showing dissipation of LD along each of the 11 cowpea chromosomes; insert on the top right corners displays genome-wide LD decay at *r*^2^ = 0.1 within 1.25 Mb between pairs of markers. For both figures, the *X*-axis is the LD measure based on correlation coefficient *r*^2^ and *Y*-axis is physical distance (Mb).

We examined LD decay within each genetic population and in the entire population, and the result is presented in Figure 5. First, the marker panel deciphered the rate of LD decay within each of the four genetic populations, with biparental RILs registering the slowest decay rate as expected, followed by multiparental RILs, while breeding lines and accessions displayed the fastest LD decay rates (Figure 5(a)). Chromosome-wide LD decay for the entire population showed variable LD decay rates on each chromosome, and when averaged across the genome, LD decayed down to *r*^2^ = 0.1 at an average distance of 1.25 Mb between pairs of markers (Figure 5(b)).

**Figure 6:**
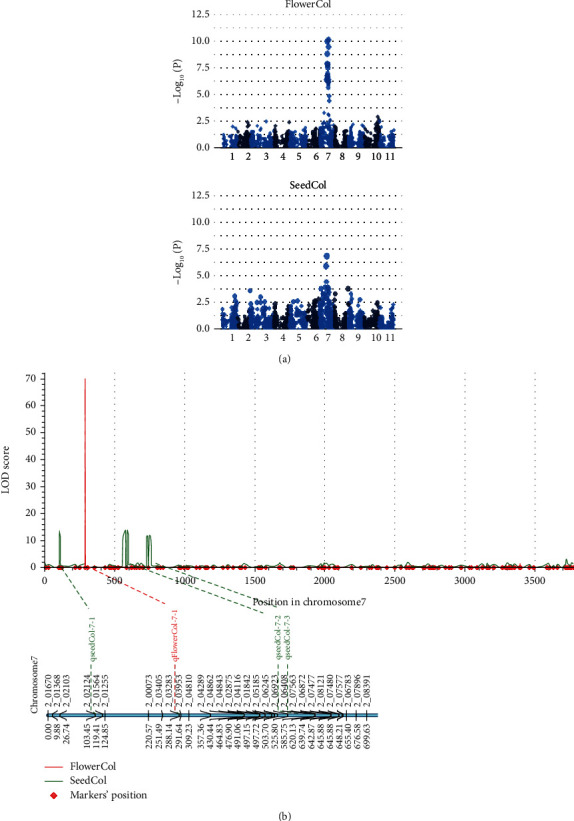
Trait mapping potential of the cowpea mid-density marker panel. (a) Genome-wide association signals for flower color and seed color traits on chromosome seven. (b) Linkage mapping showing the positions on chromosome seven where seed and flower color QTL are located.

Although the mid-density panel has a relatively small number of SNPs, we tested its potential to generate hypotheses regarding regions associated with target traits using cowpea flower and seed color. A genome-wide scan identified significant association signals for seed color and flower color, spanning a known genomic region on chromosome Vu07 responsible for pigmentations in cowpea (Figure 6(a)). These SNPs displayed moderate-to-high linkage disequilibrium, and pairwise LD (r^2^) ranged from 0.3 to 1.0 with a mean of 0.5. In addition, linkage mapping in a biparentl subset revealed a major QTL for flower color (qFlowerCol-7-1), explaining 77% of phenotypic and three minor QTLs for seed color (qseedCol-7-1, qseedCol-7-2, qseedCol-7-3) within the same region on chromosome Vu07 (Figure 6(b)).

The mapped region harbored 26 significant SNPs, three overlapping for both flower and seed color (Table 4). The peak SNPs 2_34565 and 2_06783 on chromosome Vu07 explained 18% and 12% of the variation in flower color and seed color, respectively (Table 4). These flower and seed color association signals spanned a region harboring several model genes. One of the genes in this region is *Vigun07g110700* which is a basic helix-loop-helix (bHLH) DNA-binding superfamily protein known to be involved in pigment regulation.

### 3.5. Quality Control Application in Breeding

**Figure 7:**
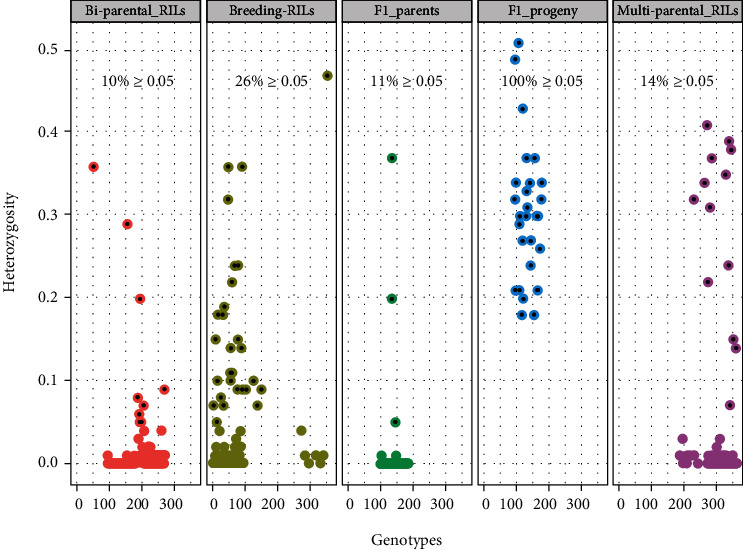
Distribution of the proportion of heterozygosity within five groups of cowpea genotypes. The dot plots depict the percentage of genotypes in each group with a heterozygosity level above 0.05. The overlaid black dots represent individuals whose heterozygosity level exceeds 0.05.

We assessed the ability of the SNP panel for QC/QA application in cowpea breeding by testing how well the marker panel can discern contamination and/or the level of genetic purity among cowpea genotypes. The *Cowpea mid-density genotyping panel V1.0.* was able to identify highly heterozygous individuals from the essentially homozygous others in each population (Figure 7). As expected, heterozygosity distribution showed F_1_s skewed towards the highest proportion relative to the other categories (Figure 7 and Supplementary Figure 3). The other four categories (parents, breeding lines, biparental, and multiparental RILs) exhibited a low proportion of heterozygosity; however, there were outliers representing heterozygous individuals in these categories. The analysis revealed that 100% of the F_1_ progenies were above the heterozygosity threshold of 0.05 (Figure 7). Among the categories that are expected to be highly homozygous and homogenous, the biparental RILs had the lowest percent (10%) of individuals with heterozygosity above 0.05, followed by the parents of F_1_ progenies (11%), multiparental RILs (14%), and breeding lines (26%).

Further, a cluster analysis revealed high genetic diversity among the parents of the 30 F_1_ progenies, with the parents being placed into three clusters (Supplementary Figure 4). Strikingly, two known IITA sister lines IT99K-573-1-1 and IT99K-573-2-1 were grouped together in cluster III. One hundred ninety-one (191) SNPs were ≥70% polymorphic between the 30 parental pairs. Further, 742 SNPs had intermediate polymorphism (50-60%) between the parental pairs, while the rest were less than 50% polymorphic. The lowest proportion (16%) of polymorphic markers was registered between parents IT15K-2241-2 and IT99K-573-2-1, while the highest (61%) was recorded between IT04K-267-8 and SANZI (Supplementary Figure 5). Using polymorphic SNPs only, the levels of hybridity of the 30 F_1_ progenies were assessed, and the distribution is presented in Supplementary Figure 6. Hybridity ranged from 23% in a cross of IT15K-2241-2 × IT99K-573-2-1 to 97% in a cross of IT97K-568-11 × IT90K-76. Overall, 40% of the F_1_s had hybridity ≥ 70%, while 57% had intermediate hybridity (30-60%) and 3% had hybridity below 30% (Supplementary Figure 6).

### 3.6. Relative Cost

Small breeding programs find genomic tools quite expensive which limits deployment. The cost of genomic applications can be economized by deploying a relatively small number of highly informative single-nucleotide polymorphisms (SNP) with even genome coverage. For cowpea, utilization of an existing 51 K iSelect array by breeding programs has been limited due to the relatively high cost. For instance, the current cost of genotyping a single experimental sample on an Infinium iSelect array with 45 K SNP panel is about $371 for a project size of 1000 samples (August 2022; https://cidr.jhmi.edu/xtras/shared/documents/pricing.pdf). For the same sample size, the cost reduces to about $107 per sample when using a 6 K SNP panel. In either case, the costs are quite high for small breeding programs in developing countries. The cost of screening samples using the 2 K *Cowpea mid-density genotyping panel V1.0*. described in the present study is about $10 per sample (September 2022; https://excellenceinbreeding.org/sites/default/files/archive/EiB%20genotyping%20services_1.pdf). For most molecular applications that do not require high-density markers, this panel is cost-effective and acceptable to most breeding programs. Note, however, that this comparison of cost ignores the substantial investment required for the development of a high-quality SNP array and that all costs quoted may vary over time.

## 4. Discussion

Crop improvement through breeding has been the major tool to lift people out of poverty and to increase the global food supply. With the projected population pressure and climate change threats, breeding must be done in a more innovative and precise way to meet the global demand for food security. This has triggered attention towards ground-breaking crop manipulation approaches in the struggle to achieve sustained increases in genetic gain. Developing and mining crop genetic and genomic resources play crucial roles in enhancing genetic gain through the maximization of diversity and the discovery of molecular tools that will accelerate breeding for traits of economic importance. Such efforts in cowpea have led to the development of genomic and genetic resources, including over 15,000 gene bank accessions [8], cowpea MAGIC population [9], and minicore populations [8, 25] in addition to elite breeding lines from breeding programs [5, 11].

Despite these resources, routine application of genomics in cowpea breeding is still limited, and this is partly attributable to the relatively high cost of existing high-density genotyping platforms. This calls for the development of cost-effective platforms that can be utilized by breeders in the developing world. To this call, Wu et al. [20] developed a low-density Kompetitive Allele-Specific PCR (KASP) SNP genotyping platform consisting of 50 informative SNPs derived from the same Cowpea iSelect Consortium Array. The authors described the low-density KASP panel as cost-effective for cowpea germplasm genetic diversity assessment and variety identification. Compared to KASP where the cost of developing assays increases with marker panel size, DArTag is still regarded as the most economical method for mid-density genotyping, where up to 4,000 markers per panel can be assayed at a cost of ~$10 per sample (https://cgspace.cgiar.org/handle/10568/122581?show=full; accessed on October 4 2022). KASP assays, on the other hand, are most suited and cost-effective for low-density genotyping (https://excellenceinbreeding.org/sites/default/files/archive/EiB%20genotyping%20services_1.pdf; accessed on October 4, 2022). The *Cowpea mid-density genotyping panel V1.0*. described in the present study has a moderate number of informative SNPs and is based on a relatively low-cost DArT platform [21]. The SNPs were carefully selected and designed to have even genome coverage [44]. The present study dissected the properties of this marker panel and its potential utility in cowpea genetic improvement.

We started by examining how informative the marker panel is by looking at the distribution of minor allele frequency (MnAF) in the entire population. About 78% (1,882) of the SNP markers in the panel had MAF above 0.2, with the remaining 22% of SNPs still having MAF above 0.05. Minor allele frequency is widely used in population genetics studies because it provides information to differentiate between common and rare variants [45, 46]. It also determines allele diversity and heritability in the population, and it has been shown that markers with high MAF have high-resolution power and are good at detecting QTL [45, 46]. The moderate-to-high MnAF observed in the present study suggested that the cowpea mid-density SNP panel is informative, making it a useful genetic resource for the cowpea scientific community.

When we scrutinized SNP distributions on each chromosome, even coverage of markers was depicted across all 11 chromosomes with an average density of 203 SNPs per chromosome and approximately one SNP every 200 Kb. This marker density and distribution are modest for the dissection of molecular diversity, genetic relatedness, population structure, linkage disequilibrium, genomic selection, and possibly a medium-resolution QTL discovery. Marker densities of less than 5,000 SNPs that are well distributed across the genome have been deployed successfully to decipher genetic diversity and other molecular and genetic applications in crops [47–49].

Population structure analysis assessed how well the panel can discern diversity and stratification in genetic populations. The genetic structure of a population is defined as a group of individuals sharing a common gene pool, and it determines its capacity to be improved or changed by selection [50]. Assessing population structure, therefore, is fundamental both in guiding breeding options and in association studies leading to trait discoveries. By using a constituted population with prior knowledge of the structure, we were able to validate the effectiveness of the marker panel in detecting population stratification. STRUCTURE analysis initially revealed two major groups, correctly isolating the biparental RILs from the rest of the groups. The fact that biparental RILs were quite distant from other populations, the separation among breeding lines and multiparental RILs was confounded; however, reanalysis excluding biparental RILs exposed the multiparental RILs as a distinct subgroup but maintained the accessions and breeding lines in the same group. This suggested three groups in the population, but the closely related biparental RILs confounded a clear population structure. Similar patterns were depicted by PCA where multiparental RILs, though less scattered, were grouped with the accessions and breeding lines, which were more diverse. DAPC revealed a clear differentiation into three gene pools. The DAPC analysis placed all the biparental RILs in a single, less scattered group, an outcome that was expected given that the biparental RILs share a wild relative's alleles from TVNu1158, as such they constituted a unique gene pool. The second gene pool consisted of the multiparental RILs that were moderately scattered, attesting to the diversity emanating from multiple numbers of parents used in developing this population. A third gene pool consisted of accessions and breeding lines. This group was the most scattered, an observation that was also expected given the inherent diversity of the breeding lines and accessions. The categorization of the breeding lines and accessions in the same group was not surprising, given that some of these accessions have been used in the breeding program to develop the breeding lines. The outcome of these analyses suggested that the mid-density panel is appropriate for genetic diversity analysis.

Population structure analysis was further corroborated by pairwise differentiation measures (*F*_ST_ and *G*_ST_) and Euclidian genetic distance between the four genetic populations, which depicted higher differentiation between the biparental RILs and the rest of the groups. These results were also supported by pairwise Euclidean genetic distances and gene flow estimates, which revealed the same pattern of genetic relationships among these four populations. Wright's *F*_ST_ [51] and Nei's *G*_ST_ [52] are statistics that measure the proportion of genetic diversity in a population [53]. These two statistics are equivalent when there are only two alleles at a locus, and in the case of multiple alleles, *G*_ST_ is equivalent to the weighted average of *F*_ST_ for all alleles [52]. Consequently, in the present study, the two statistics depicted the same differentiation pattern among populations. Some past genetic studies in cowpea have used *F*_ST_ to assess the extent of differentiation between subpopulations. For instance, Gbedevi et al. [49] reported low-to-moderate pairwise *F*_ST_ values in the range of 0.014 to 0.117 and a mean 0.072 among six subpopulations of cowpea accessions grouped by geographic regions in Togo. Using 15 SSR markers, Sarr et al. [54] reported genetic differentiation (*F*_ST_) to vary from 0.018 to 0.100 among cowpea accessions collected from different regions of Senegal. Average *F*_ST_ = 0.075 was reported among cowpea accessions collected from Ethiopia [55]. Low *F*_ST_ values (low differentiation) indicate that little variation is proportioned between populations, while high values denote that a large amount of variation is found among populations [53]. The studies mentioned above attributed the cause of observed low *F*_ST_ values to short distances between geographical regions of collection that facilitated an easy exchange of genetic materials between regions. Generally, self-pollinated crops tend to have low genetic diversity, and it has been observed that differentiation among populations of self-pollinated crops like cowpea is generally low [55–57]. In the present study, the observed high differentiation between the biparental RILs and the rest of the populations was expected given that one of the parents of the biparental RILs is a wild relative; hence, this population has a unique gene pool, which explains why it is highly differentiated from the rest of the genetic populations. Cowpea is reported to have evolved from a few progenitors, and it exhibits very limited gene flow between wild and cultivated types [58–61]. Gene flow estimates (*N*_*m*_) in the present study were high among breeding lines, multiparental RILs, and accessions (*N*_*m*_ = 0.89 to 3.9) compared to that between biparental RILs and the rest of the populations (*N*_*m*_ = 0.36 to 0.58). Upon checking pedigree records from our breeding program, it was evident that the parents of most breeding lines came from the accessions, while that of the multiparental RILs came from the elite breeding lines [9]. Indeed, multiparental RILs and the breeding lines had the highest gene flow (*N*_*m*_ = 3.9) and, strikingly, the lowest genetic distance (Dist. = 8.38) between them, confirming that these two populations share common alleles. These results implied that the *Cowpea mid-density genotyping panel V1.0.* was able to resolve the structure and diversity in the population.

Results of AMOVA further revealed higher variation among the genetic populations than between the populations. A recent study using 255 cowpea accessions collected from six regions in Togo reported significant genetic variations among and within populations, with variations among individuals that were within each of the six geographic origins explaining the highest percentage (78%) of the total variability [49]. Several authors have reported similar studies. For instance, using 671 cowpea accessions obtained from 8 regions of Senegal, variance among individuals within the regions accounted for 75% of the total variation, followed by variance within accessions (14%) and between populations (11%) [54]. Higher variations within population *vis a vis* between populations were also reported in cowpea [24, 62–64]. The higher genetic variance within populations than between populations has been explained in terms of possible gene flow between populations through germplasm sharing across geographic regions [24, 49, 54, 62, 63]. In the present study, using materials from the different genetic populations in routine breeding must have facilitated gene flow between the four groups, leading to higher genetic variance within than between the groups.

Linkage disequilibrium decay was examined in the four genetic populations and the entire population. LD decay rates varied across chromosomes, with Vu03 and Vu09 showing the lowest and fastest LD decay rates, respectively. Recombination frequency, a factor that determines LD decay rate, was found to vary along the 11 chromosomes of cowpea [1]. The pattern of this recombination rate corresponded with the chromosome-wide LD decay rates observed in the present study. Despite the moderate number of SNPs in the mid-density panel, the LD decay pattern in the population was resolvable, possibly because the set of SNPs has been carefully selected to have even genome coverage. For instance, LD displayed a slower dissipation in the biparental RILs than in the other three genetic populations. This is an expected outcome, given that biparental populations are limited in the number of genetic recombination and alleles. On the other hand, the accessions, breeding lines, and multiparental RILs have much higher recombination rates than biparental RILs [65, 66] and were correctly depicted to show faster LD decay. Overall, the genome-wide LD decay in the entire population extended to 1.25 Mb, meaning the LD between any two markers dissipated when the markers were approximately 1 Mb apart. This LD decay is moderate and is typical of self-pollinated crops with limited chances of recombination from natural out-crossing [67]. In a population of 274 cowpea accessions, using 3,127 SNPs, an LD decay rate of 100 kb, smaller than what is observed in the present study, was reported [68]. It should be noted that, in the present study, populations consisted of genetic groups combining both high and low recombination frequency backgrounds, a possible reason for the average genome-wide LD decay to extend up to ~1 Mb. Nevertheless, the LD decay rate in the present study falls in the ranges reported in the cowpea minicore population, where chromosome-wide LD varied from 809 kb (~0.8 Mb) to 4705 kb (~4.6 Mb) [12]. In asparagus bean (*Vigna. unguiculata* ssp. *sesquipedalis*), a relatively high LD of ∼1.88 Mb was reported [69]. Overall, these observations indicate that the LD decay distances are fairly long in autogamous species. In contrast, LD declines rapidly in allogamous species where physical recombination is more common. For instance, LD decays within only a few kilobases in maize [70] and only 200 bp in a wild sunflower population [71]. Despite the observed slow decay rate in cowpea populations, such populations have been used to map quantitative trait loci for several traits successfully [14, 72–75].

High-resolution QTL mapping requires high-density marker panels, a clear limitation in our mid-density SNP panel. However, the mapping of a region on chromosome Vu07, which was previously identified to harbor flower and seed coat color gene *Vigun07g110700* [72, 75], suggested that the panel may be used to hypothesize candidate QTL regions. Prior to these studies, it was reported that a lack of pigment in the flower was often associated with a lack of pigment in the seed coat, suggesting a pleiotropic effect of the gene [76]. Given the small size of this SNP panel, deployment in trait mapping would require large population size to attain the required statistical power for QTL detection. It has been demonstrated that genotyping more individuals with fewer markers is better than genotyping fewer individuals with more markers [77].

The potential of the marker panel for QC/QA in cowpea was also assessed. Our results showed the panel to excellently deduce heterogeneity within different categories of cowpea genotypes. As expected, the F_1_ progenies displayed the highest level of heterozygosity, implying they were true hybrids. Interestingly, elite breeding lines and RILs showed a low proportion of heterozygosity, and yet potentially impure individuals were detectable within each category, with the elite breeding lines exhibiting the highest percentage (26%) of heterozygous individuals at some loci. The observed high level of heterogeneity among the inbred lines suggested the need to purify these lines prior to using them as parents in the breeding program and further demonstrated that the marker panel is effective in detecting parental purity.

Knowing that the display of heterozygosity among F_1_s may not necessarily determine whether they are true hybrids, we assessed the polymorphism of markers between each parental combination of the F_1_s and used the polymorphic SNPs to authenticate hybridity. The moderate-to-high marker polymorphisms observed among the parental pairs implied that these sets of polymorphic markers would also delineate the hybridity of F_1_ progenies with high accuracy. In fact, we identified over 191 SNPs that had high polymorphism across these parental pairs. Moreover, the parents were also genetically diverse, meaning that these 191 SNPs could be considered as additional QC/QA SNPs for cowpea to the 17 KASP-based previously described for cowpea [27]. A high degree of hybridity (above 70%) was recorded in more than 40% of the F_1_s, and about 57% had intermediate hybridity; consequently, 97% of the F_1_s had moderate-to-high hybridity, and only one F_1_ progeny registered hybridity of less than 30%. A previous study on hybridity using 17 KASP-based SNPs detected 79% true F_1_s and 14% self-fertilization in a sample of 1,436 F_1_ plants [27]. Genetic purity of parental lines and hybridity authentication are important quality control criteria in breeding that directly affect the quality of lines and varieties being developed [27, 78]. Our results further demonstrate the effectiveness of this cost-efficient marker panel for genetic purity assessment and other QC needs in the breeding pipeline.

## 5. Conclusion

This study deployed the DArTag SNPs in a population of 376 cowpea genetic materials to validate the usefulness of this low-cost, medium-density marker panel for various applications in cowpea breeding. We showed that the *Cowpea mid-density genotyping panel V1.0.* contains informative SNPs distributed evenly across the 11 cowpea chromosomes. The panel revealed high polymorphisms among diverse cowpea lines, and it has a modest density of about one SNP after every 200 kb. Indeed, this mid-density marker panel displayed good potential for population structure dissection, genetic diversity assessment, and potential application as QC markers in the breeding program. Cognizant of the size, we postulated that a dissection of genomic regions governing trait variation in cowpea may be possible using this marker panel. The study further unearthed the resourcefulness of the constituted cowpea population in terms of high genetic and trait variation, which will be exploited to improve this crop. It is hoped that the findings presented here will advance the practice and knowledge of molecular marker deployment to improve economic traits in crop plants, and particularly, the application of genomic-aided breeding in cowpea.

## Figures and Tables

**Table 1:** Descriptions of the cowpea genetic materials used in the study.

Type of material	Size	Description
Breeding lines	123	Favourite breeding materials including released varieties and land races often used as parents in hybridization programs. They have high yield potentials, drought tolerance, heat tolerance, and striga resistance
Accessions	22	Favourite materials selected from the IITA mini core which are part of a world collection of cowpea germplasm. They are excellent sources of drought tolerance and aphid resistance
Multiparental lines	100	Randomly sampled from the UCR cowpea MAGIC recombinant inbred lines. Have high grain yield, early maturity, drought tolerance, striga resistance, and bacterial blight resistance [9]
Biparental lines	101	Randomly sampled from IITA recombinant inbred line segregation for aphid resistance
F_1_ progenies	30	IITA crosses combining multiple traits including high yield, resistances to striga, and bacterial blight
Total	376	

**Table 2:** Pairwise genetic distance and differentiation between four genetic populations of cowpea included in the study.

Comparison	Ec.Dist^a^	*G*_ST_^b^	*F*_ST_LB^c^	*F*_ST_UB^d^	*F*_ST_^e^	*N*_*m*_
Biparental RILs vs. accessions	20.95	0.24	0.39	0.42	0.41	0.36
Biparental RILs vs. breeding lines	19.36	0.20	0.29	0.31	0.30	0.58
Biparental RILs vs. multiparental RILs	22.26	0.27	0.39	0.41	0.40	0.38
Breeding lines vs. accessions	13.56	0.11	0.12	0.13	0.13	1.67
Breeding lines vs. multiparental RILs	8.38	0.04	0.05	0.06	0.06	3.92
Multiparental RILs vs. accessions	16.89	0.17	0.21	0.22	0.22	0.89
Number of populations	4.00					
Average no. of genotypes per population	82.50					
Number of loci	2753					
Minimum	8.38	0.04	0.05	0.06	0.06	0.36
Maximum	22.26	0.27	0.39	0.42	0.41	3.92
Average	16.90	0.17	0.24	0.26	0.25	1.30

^a^Euclidean genetic distance. ^b^Nei's differentiation measure. ^c^Lower bound confidence interval. ^d^Upper bound confidence interval. ^e^Wright's differentiation measure. *N*_*m*_ is the gene flow between populations, calculated as *N*_*m*_ = 0.25 (1 − *F*_ST_)/*F*_ST_.

**Table 3:** Analysis of molecular variance (AMOVA) showing variation within and between cowpea populations.

Source of variation	DF	SS	MS	Sigma	%Var	Phi	*P* value
Between populations	3	56010.49	18670.16	87.72	8.38	0.08	0.18
Among populations	5	13912.60	2782.52	174.17	16.64	0.18	0.01
Within genotypes	321	251915.73	784.78	784.78	74.98	0.25	0.01
Total	329	321838.82	978.23	1046.67	100.00		

DF is the degree of freedom; SS is the sum of squares; MS is the mean square; sigma is the variance; Phi is the population differentiation statistics; and *P* value is the probability.

**Table 4:** Significant SNP markers associated with flower and seed colors in cowpea on chromosome Vu07.

Trait	Marker ID	Chromosome	Pos (bp)	-Log10(p)	PVAR
FlowerCol^a^	2_34565	Vu07	23,705,735	10.14	18%
	2_47670	Vu07	20,629,436	9.94	18%
	2_47424	Vu07	24,060,891	9.45	28%
	2_17108	Vu07	20,465,839	8.82	16%
	2_01670	Vu07	20,808,628	7.87	14%
	2_19077	Vu07	22,712,648	7.77	14%
	2_18459	Vu07	21174521	7.55	13%
	2_43619	Vu07	22202114	6.95	12%
	2_55172	Vu07	20295282	6.74	12%
	2_06783^∗^	Vu07	19694195	6.70	12%
	2_12758	Vu07	23341686	6.65	12%
	2_13172	Vu07	25261206	6.38	11%
	2_47143^∗^	Vu07	17922038	6.35	11%
	2_51319^∗^	Vu07	19490375	6.32	11%
	2_14370	Vu07	25158752	6.18	11%
	2_03953	Vu07	24658559	6.13	11%
	2_12882	Vu07	24521329	6.10	11%
	2_03283	Vu07	23239607	5.66	10%
	2_04843	Vu07	25438449	4.89	8%
	2_09527	Vu07	26085154	4.44	8%

SeedCol^b^	2_06783^∗^	Vu07	19,694,195	6.86	12%
	2_47143^∗^	Vu07	17,922,038	5.96	10%
	2_51319^∗^	Vu07	19,490,375	4.44	8%
	2_54172	Vu07	15,774,834	3.84	7%
	2_12490	Vu07	24,420,693	3.83	7%
	2_19423	Vu07	25,863,610	3.74	6%

^a^Flower color. ^b^Seed color. ^∗^SNPs significantly associated with both flower and seed color. PVAR refers to percent variance explained by the SNP.

## Data Availability

The SNP data used to support the findings of this study have been deposited in the European Nucleotide Archive (ENA) database (https://www.ebi.ac.uk/ena/browser/view/PRJEB56743) under the accession number PRJEB56743 (ERP141707).

## Supplementary Materials

Supplementary 1 Supplementary Figure 1: classification of 330 cowpea genotypes by biological groups and inferred gene pool. (A) Heatmap shows the classification of the four genetic populations overlaid onto the groupings inferred by STRUCTURE. Inferred group 1 is aligned with biparental RILs, and group 2 is aligned with breeding lines, multiparental RILs, and accessions, while admixed group shows scattered alignment covering all the 4 genetic populations. (B) Dendrogram displaying grouping by genetic population information with biparental RILs clearly forming one group (red dots) while the second bigger group has a combination of breeding lines, accessions, and multiparental RILs. Supplementary Figure 2: STRUCTURE among diverse lines after excluding the biparental RILs from the rest of the populations. (A) Plot of *K* versus *DeltaK* showing the most probable number of subgroupings (*K* = 2) after excluding the biparental RILs from the population. (B) STRUCTURE bar plots showing two subgroups after excluding the biparental recombinant inbred lines (RILs). (C) Proportion of breeding lines, accessions, and multiparental RILs in the groups inferred by STRUCTURE after excluding biparental RILs. A total of 40 lines were assigned to group 1 in which the multiparental lines had zero membership. Group 2 had 158 lines, multiparental RILs forming a greater proportion of this group, followed by the breeding lines. A total of 36 lines were categorized as admixed since they had almost an equal probability of belonging to both groups. Supplementary Figure 3: box plot depicting heterozygosity dispersion within the five groups. The outliers are represented by asterisk, showing genotypes with unexpected levels of heterozygosity. Supplementary Figure 4: genetic relationship among parents used to make the 30 F_1_ crosses used in the study. An unrotated archaeopteryx tree was constructed based on the neighbor-joining algorithm in TASSEL using 2,163 DArTag-filtered SNPs with MnAF > 0.05. Inscribed Eclipse with dotted red lines highlights two IITA sister lines correctly clustered together in cluster III. Supplementary Figure 5: distribution of the extent of marker polymorphism between each pair of parents used in making the 30 F_1_ progenies included in the study. The percentage of polymorphic markers is presented on the horizontal axis and the list of parental combinations is on the vertical axis. Supplementary Figure 6: distribution of the level of hybridity of the 30 F_1_ progenies using polymorphic DArTag SNPs. Percent hybridity is presented on the horizontal axis, and the F_1_ progenies are listed on the vertical axis. The vertical red lines demarcate the level of hybridity from the lowest (hybridity < 30%) to intermediate (hybridity between 30% and 60%) to the highest (hybridity equal or above 70%).

Supplementary 2 Table 1: list of markers selected to constitute the cowpea mid-density genotyping panel V1.0. and the SNP design sequences.

Supplementary 3 Table 2: chromosome-wide proportion of heterozygosity. MAF: major allele frequency; MnAF: minor allele frequency; missing data.

Supplementary 4 Table 3: chromosome-wide SNP density and distance between SNPs.

Supplementary 5 Table 4: assignment of 330 cowpea lines into groups by STRUCTURE analysis.
