# Supplementary material for: A Mid-Density Single-Nucleotide Polymorphism Panel for Molecular Applications in Cowpea (Vigna unguiculata (L.) Walp)
Source: Int J Genomics. 2024 Jan 9;2024:9912987. doi: 10.1155/2024/9912987 (PMC10791481; doi:10.1155/2024/9912987)
Supplement: Supplementary 1 — Supplementary Figure 1: classification of 330 cowpea genotypes by biological groups and inferred gene pool. (A) Heatmap shows the classification of the four genetic populations overlaid onto the groupings inferred by STRUCTURE. Inferred group 1 is aligned with biparental RILs, and group 2 is aligned with breeding lines, multiparental RILs, and accessions, while admixed group shows scattered alignment covering all the 4 genetic populations. (B) Dendrogram displaying grouping by genetic population information with biparental RILs clearly forming one group (red dots) while the second bigger group has a combination of breeding lines, accessions, and multiparental RILs. Supplementary Figure 2: STRUCTURE among diverse lines after excluding the biparental RILs from the rest of the populations. (A) Plot of K versus DeltaK showing the most probable number of subgroupings (K = 2) after excluding the biparental RILs from the population. (B) STRUCTURE bar plots showing two subgroups after excluding the biparental recombinant inbred lines (RILs). (C) Proportion of breeding lines, accessions, and multiparental RILs in the groups inferred by STRUCTURE after excluding biparental RILs. A total of 40 lines were assigned to group 1 in which the multiparental lines had zero membership. Group 2 had 158 lines, multiparental RILs forming a greater proportion of this group, followed by the breeding lines. A total of 36 lines were categorized as admixed since they had almost an equal probability of belonging to both groups. Supplementary Figure 3: box plot depicting heterozygosity dispersion within the five groups. The outliers are represented by asterisk, showing genotypes with unexpected levels of heterozygosity. Supplementary Figure 4: genetic relationship among parents used to make the 30 F1 crosses used in the study. An unrotated archaeopteryx tree was constructed based on the neighbor-joining algorithm in TASSEL using 2,163 DArTag-filtered SNPs with MnAF > 0.05. Inscribed Eclipse with [file 9912987.f1.docx]

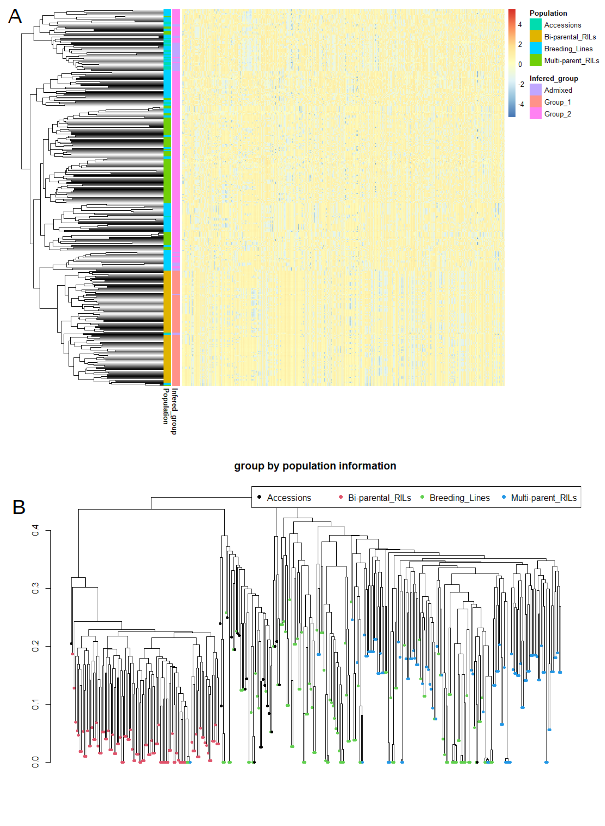
**Supplementary Figure 1** Classification of 330 cowpea genotypes by biological groups and inferred gene pool. (A) Heatmap shows the classification of the four genetic populations overlaid onto the groupings inferred by STRUCTURE. Inferred group 1 is aligned with bi-parental RILs, group 2 is aligned with breeding lines, multi-parental RILs and accessions while admixed group shows scattered alignment covering all the 4 genetic populations. (B) Dendrogram displaying grouping by genetic population information with bi-parental RILs clearly forming one group (red dots) while the second bigger group has a combination of breeding lines, accessions and multi-parental RILS


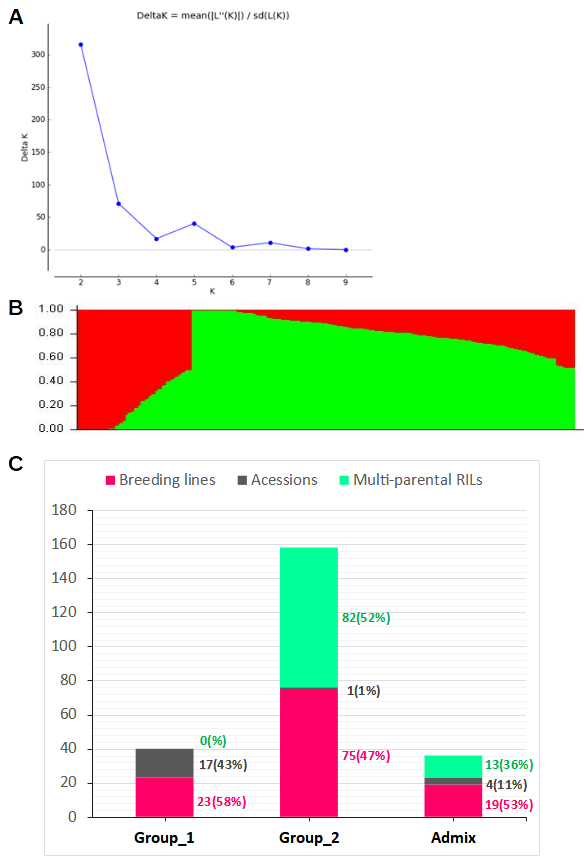


Accession

**Supplementary Figure 2** STRUCTURE among diverse lines after excluding the bi-parental RILs from the rest of the populations. (A) Plot of *K* versus *DeltaK* showing the most probable number of sub-groupings (K=2) after excluding the bi-parental RILs from the population (B) STRUCTURE bar plots showing two sub-groups after excluding the bi-parental recombinant inbred lines (RILs). (C) Proportion of breeding lines, accessions and multi-parental RILs in the groups inferred by STRUCTURE after excluding bi-parental RILs. A total of 40 lines were assigned to group 1 in which the multi-parental lines had zero membership. Group 2 had 158 lines, multi-parental RILs forming a greater proportion of this group, followed by the breeding lines. A total of 36 lines were categorized as admixed since they had almost equal probability of belonging to both groups.


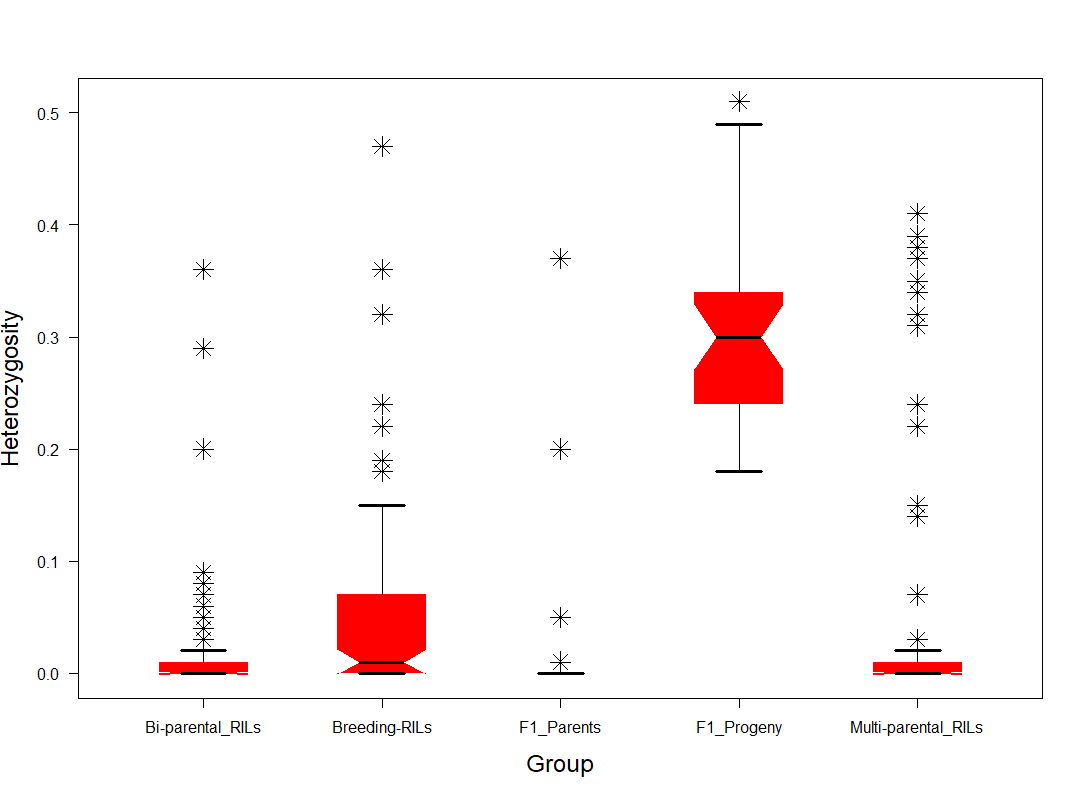


**Supplementary Figure 3** Box plot depicting heterozygosity dispersion within the five groups. The outliers are represented by Asterix, showing genotypes with unexpected levels of heterozygosity.


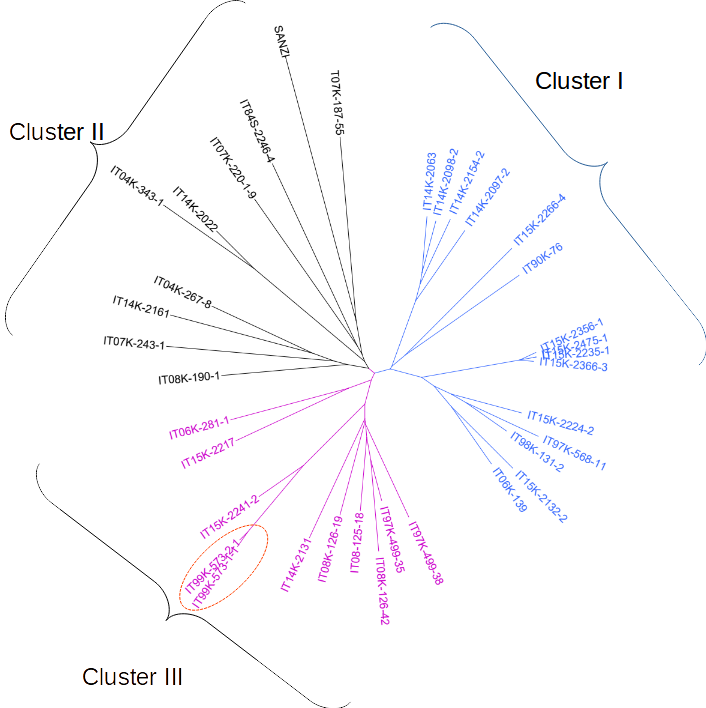


**Supplementary Figure 4** Genetic relationship among parents used to make the 30 F1 crosses used in the study. Unrotated archaeopteryx tree constructed based on Neighbour-joining algorithm in TASSEL using 2,163 DArTag filtered SNPs with MnAF >0.05. Inscribed Eclipse with dotted red lines highlights two IITA sister lines correctly clustered together in cluster III.


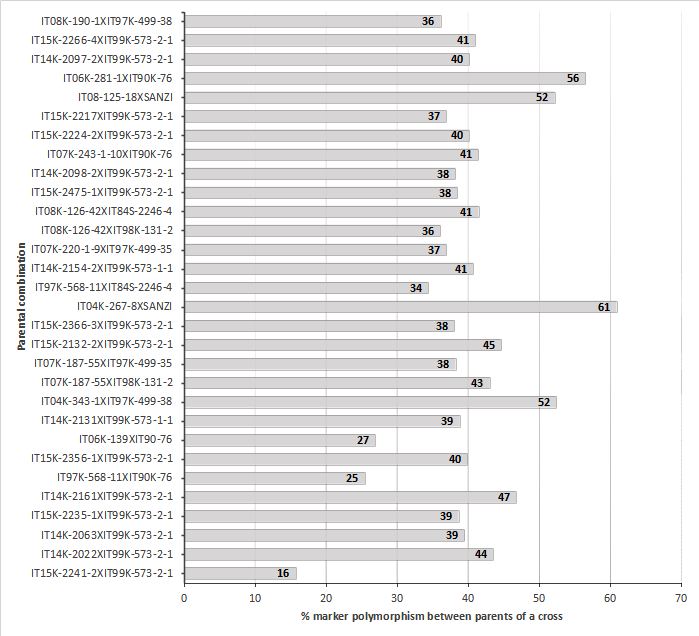


**Supplementary Figure 5** Distribution of the extent of marker polymorphism between each pair of parents used in making the 30 F_1_ progenies included in the study. The percentage of polymorphic markers is presented on the horizontal axis, and the list of parental combinations is on the vertical axis.


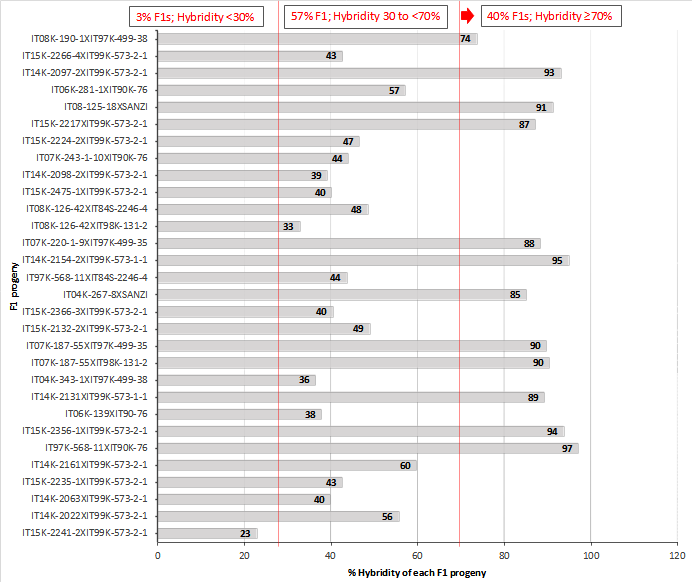


**Supplementary Figure 6** Distribution of the level of hybridity of the 30 F1 progenies using polymorphic DArTag SNPs. Percent hybridity is presented on the horizontal axis, and the F1 progenies are listed on the vertical axis. The vertical red lines demarcate the level of hybridity from the lowest (hybridity <30%) to intermediate (hybridity between 30% to 60%) to the highest (hybridity equal or above 70%).
