# Supplementary material for: A Mid-Density Single-Nucleotide Polymorphism Panel for Molecular Applications in Cowpea (Vigna unguiculata (L.) Walp)
Source: Int J Genomics. 2024 Jan 9;2024:9912987. doi: 10.1155/2024/9912987 (PMC10791481; doi:10.1155/2024/9912987)
Supplement: Supplementary 3 — Table 2: chromosome-wide proportion of heterozygosity. MAF: major allele frequency; MnAF: minor allele frequency; missing data. [file 9912987.f3.docx]

**Supplementary Table 2** Chromosome-wise proportion of heterozygosity, Major allele frequency (MAF), Minor Allele Frequency (MnAF), and missing data.

| Chr | Heterozygosity | MAF | MnAF | Missing |
| --- | --- | --- | --- | --- |
| VU01 | 0.059 | 0.69 | 0.31 | 0.055 |
| VU02 | 0.059 | 0.65 | 0.28 | 0.164 |
| VU03 | 0.047 | 0.64 | 0.21 | 0.239 |
| VU04 | 0.048 | 0.75 | 0.25 | 0.051 |
| VU05 | 0.065 | 0.67 | 0.33 | 0.049 |
| VU06 | 0.057 | 0.71 | 0.29 | 0.033 |
| VU07 | 0.053 | 0.69 | 0.31 | 0.054 |
| VU08 | 0.061 | 0.68 | 0.32 | 0.035 |
| VU09 | 0.055 | 0.71 | 0.29 | 0.047 |
| VU10 | 0.055 | 0.73 | 0.27 | 0.045 |
| VU11 | 0.057 | 0.69 | 0.31 | 0.049 |
| Min | 0.047 | 0.64 | 0.21 | 0.03 |
| Max | 0.065 | 0.75 | 0.33 | 0.24 |
| Mean | 0.056 | 0.69 | 0.29 | 0.07 |
