# Supplementary material for: A Mid-Density Single-Nucleotide Polymorphism Panel for Molecular Applications in Cowpea (Vigna unguiculata (L.) Walp)
Source: Int J Genomics. 2024 Jan 9;2024:9912987. doi: 10.1155/2024/9912987 (PMC10791481; doi:10.1155/2024/9912987)
Supplement: Supplementary 4 — Table 3: chromosome-wide SNP density and distance between SNPs. [file 9912987.f4.docx]

**Supplementary Table 3** Chromosome-wise SNP density and distance between SNPs

| Chr | Start-End pos (bp) | Length (Mb)^a^ | | SNPs/Chr^b^ | Density^c^ | SNPs Dist(Mb)^d^ |
| --- | --- | --- | --- | --- | --- | --- |
| VU01 | 78762-42014104 | 41.94 | | 188 | 4.48 | 0.22 |
| VU02 | 71029-33817793 | 33.75 | | 169 | 5.01 | 0.2 |
| VU03 | 255395-65242470 | 64.99 | | 295 | 4.54 | 0.22 |
| VU04 | 351398-42555590 | 42.2 | | 191 | 4.53 | 0.22 |
| VU05 | 216823-48682333 | 48.47 | | 188 | 3.88 | 0.26 |
| VU06 | 124432-34261190 | 34.14 | | 197 | 5.77 | 0.17 |
| VU07 | 51380-40659215 | 40.61 | | 256 | 6.3 | 0.16 |
| VU08 | 126058-38248903 | 38.12 | | 193 | 5.06 | 0.2 |
| VU09 | 38839-43850276 | 43.81 | | 219 | 5 | 0.2 |
| VU10 | 72119-41297765 | 41.23 | | 165 | 4 | 0.25 |
| VU11 | 50894-41324185 | 41.27 | | 169 | 4.09 | 0.24 |
| Min |  | 33.75 | | 165 | 3.88 | 0.16 |
| Max |  | 64.99 | | 295 | 6.3 | 0.26 |
| Mean |  | 42.77 | | 202.73 | 4.79 | 0.21 |
| ^a^Chromosome length in million base pairs | | | |  |  |  |
| ^b^Number of SNPs per chromosome | | | |  |  |  |

^c^SNP Density computed as the ratio of the number of SNPs to the chromosome length (Number of SNPs per unit Mb)

^d^Average distance between SNPs computed as the ratio of chromosome length to the number of SNPs
