# Supplementary material for: A Mid-Density Single-Nucleotide Polymorphism Panel for Molecular Applications in Cowpea (Vigna unguiculata (L.) Walp)
Source: Int J Genomics. 2024 Jan 9;2024:9912987. doi: 10.1155/2024/9912987 (PMC10791481; doi:10.1155/2024/9912987)
Supplement: Supplementary 5 — Table 4: assignment of 330 cowpea lines into groups by STRUCTURE analysis. [file 9912987.f5.docx]

**Supplementary Table 4** Assignment of 330 cowpea lines into groups by STRUCTURE analysis

|  |  |  | **The proportion of cowpea lines within each inferred group** | | | |
| --- | --- | --- | --- | --- | --- | --- |
| **Group** | **No. Lines** | **% Lines** | **No. (%) Breeding lines** | **No. (%) Bi-parental RILs** | **No. (%) Multi-parental RILs** | **No. (%) Accessions** |
| Group 1 | 98 | 30 | 2(2%) | 95(97%) | 1(1%) | 0(0%) |
| Group 2 | 195 | 59 | 90(46%) | 0(0%) | 94(48%) | 11(6%) |
| Admixed | 37 | 11 | 25(68%) | 1(3%) | 0(0%) | 11(30%) |
| Total | 330 | 100 | 117 | 96 | 95 | 22 |
